# Seroprevalence of Cytomegalo Virus (CMV) among pregnant women in Thika, Kenya

**DOI:** 10.1186/1756-0500-7-794

**Published:** 2014-11-12

**Authors:** Zakayo Maingi, Anthony Kebira Nyamache

**Affiliations:** Department of Microbiology, Kenyatta University, P.O. Box 43844, 00100 Nairobi, Kenya; Department of Medical Laboratory Science, Kenyatta University, Nairobi, Kenya

## Abstract

**Background:**

The fetal consequences of CMV infection have made it one of the most serious infections contracted during pregnancy. Despite the posed teratogenic risk during pregnancy, there is no national screening test for CMV infection is available during pregnancy in Kenya. Thus little is known on its epidemiological data that is necessary for health planners and care providers.

**Methods:**

A cross sectional study was conducted at Thika district level 5 hospital, Kenya to investigate seroprevalence of CMV infections and associated possible risk factors among pregnant women. Structured questionnaires were used to gather socio-demographic data and ELISA was used to detect CMV infections using IgG and IgM.

**Results:**

Out of 260 pregnant women, 201 (77.3%) were CMV IgG 21(8.1%) CMV IgM being on acute stage of the disease. Marital status (OR = 3.7533, 95% CI =3.0231-6.9631, P < 0.0001), parity (OR = 3.7533, 95% CI = 3.0231-6.9631, P < 0.0001), and education (OR = 3.7533, 95% CI = 3.0231-6.9631, P < 0.0001), history of blood transfusion (OR = 0.0374, 95% CI = 0.00120-0.1168, OR = 0.3804) were found to significantly influence seropostivity in univariate analysis.

**Conclusion:**

The 88.4% CMV prevalence rate being detected among pregnant women calls for vaccine and routine screening for CMV infections and its associated risk factors in this kind of settings.

## Background

Cytomegalovirus (CMV) is the commonest among viral infections during perinatal period that cause congenital CMV infections [1]. Its clinical manifestations range from asymptomatic forms (90% of cases) to severe fetal damage that may include permanent hearing, vision loss, neurological impairment and, in rare cases, death due to abortion [2–4]. Previous studies have confirmed that CMV infection is relatively common among women of reproductive age with seroprevalence ranging from 45% to 100%. African continent like South America and Asia has one of the highest prevalence of CMV [4]. From the previous studies conducted in Africa, CMV prevalence rates in Egypt were found to be 96% [5], 85.7% Tanzania [6], 97.2% Benin [7] and 86.4% South Africa [8].

CMV is transmitted from person-to-person via close non-sexual contact, sexual activity, breastfeeding, blood transfusions, and organ transplantation [9]. For pregnant women, important sources of infection include sexual activity and contact with the urine or saliva of young children, especially their own children [10]. However, seroprevalence varies greatly with a variety of epidemiological factors such as geographical distribution, socio-economic status, marital status and parity [11].

Like other herpes viruses, primary infection is followed with established of lifelong latent infection from which periodic reaction is common [12]. At this stage, symptoms are usually absent including during reaction [13].

As far as prevention is concerned, in addition to health education campaigns, the serological screening of pregnant women has been proposed. However, there is no consensus in the scientific community concerning the implementation of screening and it is not recommended by any public health system despite its teratogenic effects because of its cost/benefit ratio [14]. However, other countries Israel, Belgium, and France their doctors do test their pregnant patients an intervention to CMV infections that should be adopted by all [14].

There is no published data concerning CMV seroprevalence in pregnant women in Kenya. The basic data concerning CMV infections during pregnancy is important for health planners and care providers. This study was therefore aimed at determining the seroprevalence, associated possible risk factors for CMV infections among pregnant women in Thika Kiambu County.

## Methods

To determine the seroprevalence of CMV among the expectant mothers, seeking health services at Thika level 5 hospitals. Consenting pregnant women were approached to participate in the study. A questionnaire was administered and socio-demographic data and blood samples were collected from 260 consenting participants during the period of Sept 2012 to April 2013. The participants were women aged between (18) eighteen years and (45) forty five years old. The participants were sampled from the antenatal clinic. A 5ml blood specimen was obtained for each subject for the evaluation of CMV serum immunoglobulin G antibody using a commercial enzyme-linked immunosorbent assay (Wampole; Inverness Medical Professional Diagnostics) in accordance with the manufacturer’s instructions.

Quantitative analysis for CMV (IgG and IgM) was performed, and the assay result interpreted as IU/mL. The manufacturer’s instructions were followed for the cut off points, which was <9 IU/mL for CMV IgG and IgM. In cases where samples were tested positive for both IgG and IgM, they further evaluated for the avidity of IgG antibodies. From the analysis, samples that were found to have an avidity index >35% were 43(79.63%) while those detected with <35% were 11(20.37%) indicated IgG, IgM predominance respectively, Table 1.

**Table 1:** **Avidity index among participants with both IgM and IgG antibodies**

Avidity index	Participants(n)	%
**<35%**	11	20.37
**>35%**	43	79.63
**Total**	**54**	**100.0**

### Statistics

Univariate and multivariate Odd ratio analysis was conducted and on CMV seropostivity for IgG and IgM and its association between IgG, IgM seropostivity and, high parity >4 deliveries, marital status, history of blood transfusion, HIV status, illiteracy, occupation, and residing location determined and *p* values less than 0.05 were considered statistically significant.

## Results

### Socio-demographic characteristics

A total of 260 pregnant women of the age group of 18–45 years and in their first or second trimesters were enrolled into this study. The mean age of the participants was 28years with most of the responds being married 168(64.61%) with those divorced being the least 27(10.39%). More than third (39.4%) of these women were literate and either in business 68(26.2%) or employed 70(26.9%). One hundred and thirty seven 137 (53.08%) of the respondents had 1–4 children with most of the participants residing in urban centres 149 (57.3%), Table 2.

**Table 2:** **Socio-demographic characteristics and associated factors with CMV infection of pregnant women in Thika, Kenya**

Variable	Participants (n/%)	IgM (n/%)	IgG (n/%)	Univariate	Multivariate
**Age group:**					
21-25	52(20)	31(12)	7(22.26)		
26-30	95(36)	5(13.68)	77(81.05)	OR = 0.0898	3.3866
31-35	54(21)	2(3.7)	46(85.19)	95% CI = 0.0017-4.4637	0.0665-172.5
36-40	18(7)	4(22.22)	10(55.56)	P = 0.2310	0.5430
41-45	10(4)	1(10)	9(90)		
**Subtotal**	**260**	**21(8.1)**	**201(77.1)**		
**Marital status:**					
Single	66(24.39)	9(13.6)	38(57.6)	OR = 3.7533	0.3897
Married	168(64.61)	12(7.2)	137(82)	95% CI =3.0231-6.9631	0.004-0.0451
Divorced	27(10.39)	0(0)	26(96.3)	P < 0.0001	0.0004
**Sub total**	**260**	**21**	**201**		
**Parity:**					
None	117(45)	12(10.35)	76(65.5)	OR = 0.2373	0.2224
One to four	138(53.08)	8(5.8)	121(87.7)	95% CI =0.1246-0.4519	0.1143-0.5643
>four	5(13.59)	0(0)	4(66.7)	P < 0.0001	P = 0.0003
**Sub total**	**260**	**20**	**240**		
**Trimester of pregnancy:**					
First	131(50.4)	9(6.9)	101(77.1)	OR = 1.881	2.3346
Second	129(49.6)	12(9.3)	100(77.5)	95% CI = 0.4448-2.6617	0.8996-3217
**Sub total**	**260**	**21**	**201**	P = 0.8532	0.7756
**Location:**					
rural	111(42.7)	9(8.1)	84(75.7)	OR = 0.8509	1.0074
urban	149(57.3)	12(8.1)	117(78.5)	95% CI = 0.4746-1.5256	0.489-2.484
**Sub total**	**260**	**21**	**201**	P = 0.5878	0.9873
**Education:**					
None	3(1.2)	0(0)	3(100)	OR = 0.6364	0.427
Primary	12(4.6)	4(33.33)	8(66.7)	95% CI = 0.0318-12.7301	0.0132-13820
Secondary	79(330.4)	9(11.4)	58(73.4)	P = 0.7675	0.1001
Tertiary	166(63.8)	8(4.8)	132(79.5)		
**Sub total**	**260**	**21**	**201**		
**Occupation:**					
Business	68(26.2)	5(7.4)	55(80.9)		
Employed	70(26.9)	3(4.3)	58(82.9)	OR 0.6369	1.6143
Housewife	33(12.7)	5(15.2)	26(78.8)	95% CI = 0.2210-1.8359	0.2047-12.7299
Farmer	43(16.5)	3(7.0)	34(79.1)	P = 0.04036	0.6494
Student	46(17.7)	5(10.9)	28(60.9)		
**Sub-total**	**260**	**21**	**201**		
**History of blood transfusion:**					
No	243(93.5)	20(8.2)	20(8.2)	OR = 0.0374	2.7125
Yes	17(6.5)	1(5.9)	12(70.6)	95% CI = 0.00120-0.1168	0.0132-0.01382
Sub Total	260	21	32	P < 0.001	P < 0.003
**HIV status:**					
positive	27(10.3)	1(3.7)	23(85.2)	OR = 0.3804	1.7767
negative	233(89.6)	20(8.6)	178(76.4)	95% CI = −0.0491-2.9448	0.5890-5.3589
**Sub total**	**260**	**21**	**201**	P = 0.3546	0.3076

*Abbreviations*: *OR* Odds Ratio, *CI* confidence interval.

### CMV seroprevalence

Out of a total of 260 pregnant women under this study, 201(77.3%) and 21(8%) had seropositive CMV IgG and IgM, respectively (Table 2). Those on the age group between 31–35 year old (54), had the highest IgG seropositive rates IgG 46 (85.19%) while those under age 21–25 years old 52 had the least 7(22.26%). However those who were HIV positive (27), had 23(85.2%) IgG seropositive, Table 2.

### Risk factors for CMV infections

Multivariate and univariate analysis was used for CMV IgG and IgM seropositive groups as dependent variable and socio-demographic variables as independent variables. P value <0.05 was considered significant. We therefore determined if age, high parity >4 deliveries, marital status, history of blood transfusion, HIV status, illiteracy, occupation, and residing location had any significance risk to predict CMV infections. However, marital status, high parity, history of blood transfusion and age were significant risk factors for CMV infection. Geographical location and occupation and HIV status were not significantly associated with CMV infection, Table 2.

## Discussion

This is the first published data on the epidemiology of CMV infections among pregnant women in Kenya. Equally few studies have been conducted among pregnant women with most studies being among blood donors. However, in this study, seroprevalence of CMV IgG 77.3% and IgM 8.1% were detected. These findings were similar to those obtained in Sudan (77.2%) [15]. Contrary to previous studies conducted in Africa, higher rates have been reported, in Benin (97.2%) [7], Egypt (96%) [16], Gambia (87%) [17], South Africa (86.4%) [18], Nigeria (100%), [19], 87% [17] Dares Salaam, Tanzania [6] and also in South East Asia [20]. However, in some of European countries, low CMV infection rates have been reported, Australia (56.9%) and France (46.8%) [21]. The low prevalence rates could be due to the inclusion of CMV screening among the antenatal profile tests and better hygienic standards [22]. The low prevalence rates of CMV in this study compared to the rest of the studies in African countries, could be due to diverse HIV infections (which is an important coinfections with CMV) [23], diverse socio-demographics, diverse cultures, population behaviour, child cares, breast feeding and sexual activity [24]. The detected 77.3% of CMV infections showed that these women were at high risk of CMV infections.

From this study, we determined the risk factors that could influence HIV infections to CMV infections. From the analysis, women who were married, aged or with high parity, were found to be at higher risk for CMV infection (Table 2). These risk factors were similar to those found by previous studies [15]. These factors increased susceptibility to acquisition of CMV infection, perhaps through the direct contact with contagious secretions from their own children or poor hygiene practiced by these women [25, 26]. In addition, in these settings, most women are usually married based on the customs of most African settings with high number of children.

There is a lot of debate concerning maternal age and CMV infection; however most studies including this study have shown elderly women to be at higher risk of CMV infection [27], while others reporting contrary [7, 18, 27]. However, other factors like geographical location, education and occupation are not significantly associated with CMV infection.

CMV IgG avidity assay seems to be one of the most accessible tools to differentiate between primary from non-primary CMV infection [28]. This technique is less expensive and it could be used to confirming CMV primary infections without the use of sophiscated polymerase chain reactions. In our study, high CMV IgG avidity were confirmed implying that in these women, their pregnancy could be maintained with a lower risk of transmitting CMV infections to their offspring (Table 1) [29]. However, this study was limited with failure to confirm CMV infections by Polymerase Chain Reaction (PCR) including failure to make a follow up of IgM seropostivity women to ascertain their infection status/seroconversion.

## Conclusion

This study shows the prevalence of 77.3% similarly to those obtain from other countries with those married aged and with high parity being at a high risk to CMV infections. This study concurs with previous studies that have suggested all women of the child bearing age to be incorporated in routine antenatal screening profile.

### Ethical

This study was approved by Kenyatta University Ethical review committee.
